# Epidermal growth factor receptor expression escapes androgen regulation in prostate cancer: a potential molecular switch for tumour growth

**DOI:** 10.1038/sj.bjc.6605376

**Published:** 2009-11-03

**Authors:** A M Traish, A Morgentaler

**Affiliations:** 1 Departments of Biochemistry and Urology, Boston University School of Medicine, Boston, MA, USA; 2 Division of Urology, Men's Health Boston, Beth Israel Deaconess Medical Center, Harvard Medical School, Boston, MA, USA

**Keywords:** androgen signalling, epidermal growth factor receptor, prostate cancer, tumour growth, metastasis, molecular switch

## Abstract

Androgen deprivation therapy reduces prostate cancer (PCa) tumour growth; however, disease relapse often ensues independently of androgen stimulation, producing androgen-refractory tumours with increased invasion, proliferation, and malignancy. Androgens downregulate epidermal growth factor receptor (EGFR) in normal prostate but not in PCa. Thus, loss of EGFR regulation and altered signalling may, in part, explain the transition of prostate tumours from androgen dependent to androgen independent. Studies in animal models, PCa cell lines, and tumour specimens suggest that androgens modulate prostate growth and function through mechanisms that involve ‘cross-talk’ between androgen receptor (AR) and growth factor receptor signalling pathways. The objective of this review is to discuss the paradoxical relationship between androgen regulation of EGFR in normal prostate and PCa. We reviewed the literature from mid-1980s through 2009 to assess the relationship between androgens and EGFR function in modulating the growth of normal prostate and PCa. Loss of androgen regulation of EGFR in PCa may be responsible for increased tumour growth, invasion, and metastasis, with important implications on the clinical management of PCa. We advance the hypothesis that a molecular switch, responsible for downregulating EGFR expression by androgens in the normal prostate, is either lost or modified in PCa.

## Cross-talk between androgen receptors and EGFR signalling pathways in prostate

**Figure 1:**
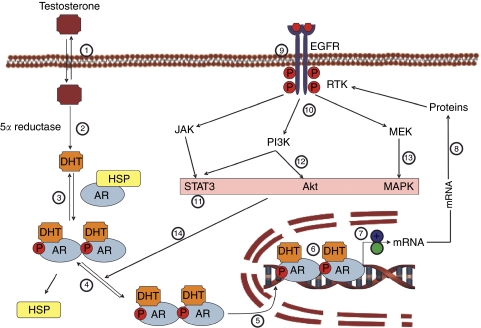
Androgen-receptor signalling in normal prostate and PCa. Free (unbound) testosterone crosses the plasma membrane phospholipid layer presumably by simple diffusion (reaction 1). Once inside the prostate cell, testosterone undergoes metabolism by the 5*α*-reductase enzyme to produce the more potent androgen 5*α*-DHT (reaction 2). 5*α*-DHT binds to the androgen receptor (AR) and causes the AR to undergo activation and transformation, which involves dissociation from heat-shock proteins and conformational (dimerisation) and biochemical changes such as phosphorylation (reaction 3 and 4). The activated and transformed 5*α*-DHT.AR complex translocates into the nucleus (reaction 5) and interacts with the androgen response element. The 5*α*-DHT.AR complex binds to the specific DNA response elements (reaction 6) leading to recruitment of coactivators or corepressors to regulate gene expression (reaction 7). We postulate that in the normal prostate, the activation of AR results in downregulation of the expression of the epidermal growth factor receptor (EGFR) mRNA (reaction 7), resulting in reduced EGFR protein synthesis (reaction 8) and the ultimate reduction of the active functional protein (reaction 9). In PCa, we propose that either a molecular switch is turned off along this pathway, probably in regulating gene expression (reaction 7; denoted by +blue colour), resulting in increased mRNA synthesis and increased protein synthesis and increased density of the functional EGFR (reactions 8 and 9). Further, in PCa, activation of EGFR by EGF results in signalling through a host of biochemical pathways (reactions 10–13), which result in activation of AR even in the absence of 5*α*-DHT. Such a switch in EGFR expression and functional activity results in tumour androgen independence. The loss of androgen regulation concomitant with increased EGFR expression or signalling in PCa through the PI3K and MAPK pathways results in androgen independence of tumour growth. These signalling pathways may activate the AR without ligand, culminating in androgen-receptor signalling, leading to cellular proliferation, migration, and survival. The references for the reactions cited in Figure 1 are as follows: Reaction 1, Heinlein and Chang, 2004; Reaction 2, Heinlein and Chang, 2004; Reaction 3, Heinlein and Chang, 2004; Reaction 4, Heinlein and Chang, 2004; Reaction 5, Heinlein and Chang, 2004; Reaction 6, Heinlein and Chang, 2004; Reaction 7, Brass *et al*, 1995; Ravenna *et al*, 1995; Itoh *et al*, 1998; Schwartz *et al*, 1998; Heinlein and Chang, 2004; Hammarsten *et al*, 2007; Pignon *et al*, 2009; Reaction 8, Nishi *et al*, 1996; Reaction 9, Traish and Wotiz, 1987; St-Arnaud *et al*, 1988; Reaction 10, Sugita *et al*, 2004; Gregory *et al*, 2005; Migliaccio *et al*, 2006; Léotoing *et al*, 2007; Zhu and Kyprianou, 2008; Recchia *et al*, 2009; Reaction 11, Migliaccio *et al*, 2006; Léotoing *et al*, 2007; Zhu and Kyprianou, 2008; Recchia *et al*, 2009; Reaction 12, Migliaccio *et al*, 2006; Léotoing *et al*, 2007; Zhu and Kyprianou, 2008; Recchia *et al*, 2009; Reaction 13, Migliaccio *et al*, 2006; Léotoing *et al*, 2007; Zhu and Kyprianou, 2008; Recchia *et al*, 2009; Reaction 14, Migliaccio *et al*, 2006; Léotoing *et al*, 2007; Zhu and Kyprianou, 2008; Recchia *et al*, 2009. Abbreviations: AKT, protein kinase B; AR, androgen receptor; ARE, androgen response element; 5*α*-DHT, dihydrotestosterone; JAK, Janus kinase; MAPK, mitogen-activated protein kinase; MEK, upstream kinases of mitogen-activated protein kinases; mRNA, messenger RNA; P13K, phosphatidylinositol 3 kinase; STAT3, signal transducer and activator of transcription 3; TK, tyrosine kinase. The colour reproduction of this figure is available on the html full text version of the manuscript.

Androgens modulate prostate growth and function through a multi-step mechanism, which involves metabolism of testosterone (T) into 5*α*-dihydrotestosterone (5*α*-DHT) by the enzyme 5*α*-reductase. The more potent 5*α*-DHT binds to the androgen receptor (AR). The ligand-bound AR complex undergoes molecular changes known as ‘activation and transformation’, resulting in specific interactions between the AR hormone complex and the specific DNA enhancer element, known as androgen response elements thought to be present in androgen-regulated genes (Figure 1).

These complex interactions lead to upregulation of androgen-specific target genes and downregulation of other androgen-specific target genes to maintain homeostasis. Among the specific target genes that are downregulated by androgen in normal prostate tissue is the EGFR. Regulation of EGFR expression by androgens is shown to be at the transcriptional level, both in normal prostate tissue and prostate cancer (PCa) cell lines (Brass *et al*, 1995; Ravenna *et al*, 1995; Nishi *et al*, 1996; Itoh *et al*, 1998; Schwartz *et al*, 1998; Hammarsten *et al*, 2007; Pignon *et al*, 2009). This regulation seems to be negatively regulated in normal cells and upregulated in PCa, especially in androgen-independent PCa. This suggests a defect in the molecular mechanism of action of androgens in PCa.

The EGFR protein binds the epidermal growth factor (EGF) and plays an important role in regulating cellular growth and function (Traish and Wotiz, 1987; St-Arnaud *et al*, 1988; Nishi *et al*, 1996; Itoh *et al*, 1998; Migliaccio *et al*, 2006; Bonaccorsi *et al*, 2004, 2007; Léotoing *et al*, 2007). Binding EGF to EGFR modulates cellular function by activating EGFR through autophosphorylation, which results in a downstream cascade that leads to increased cellular proliferation (Migliaccio *et al*, 2006). EGFR signalling results in activation of phosphoinositol 3 kinase (PI3 kinase). The latter activates the Akt family of kinases and signal transducer and activator of transcription (STAT), resulting in downstream events that regulate cellular proliferation, survival, and migration (Bonaccorsi *et al*, 2004, 2007; Migliaccio *et al*, 2006; Léotoing *et al*, 2007) (Figure 1). Moreover, these pathways activate the AR through phosphorylation in the absence of the androgen ligand, thus promoting further cellular growth without androgen stimulation. This signalling pathway, mediated via EGFR “cross-talk” with the AR pathway has an important function in regulating cell growth (Bonaccorsi *et al*, 2004, 2007; Migliaccio *et al*, 2006; Léotoing *et al*, 2007). Evidence for such ‘cross-talk’ has been observed in studies with androgen-dependent and androgen-refractory prostate tumours.

Several studies have suggested that mutations in AR coupled with disruptions in growth factor-activated pathways modulate AR signalling and may be involved in the pathology of progression to androgen-independent PCa (Scher *et al*, 1995). A large number of androgen-refractory prostate tumours retain AR expression, indicating potential activation of AR even in the absence of androgens.

The relationship between androgens and PCa has been the subject of intensive investigation and the literature is replete with studies suggesting that androgens have a crucial function in promoting PCa growth. This made androgen deprivation therapy (ADT) the mainstay for treatment of advanced PCa. However, ADT is of limited benefit because of disease relapse and devastating adverse effects of androgen deprivation, especially on the cardiovascular system (Lu-Yao *et al*, 2008). Recent epidemiological and clinical studies suggested that there is no association between T levels and risk of PCa (Roddam *et al*, 2008). There is no evidence to date to suggest that low plasma T levels are protective against PCa and that physiological levels of T increase the risk of PCa. Interestingly, T levels decline with age and PCa incidence increases with age, suggesting that a low T level may contribute to the development of cancer and normal physiological T levels may be protective against PCa (Prehn, 1999). This concept is difficult to grasp in the light of myriad studies linking PCa growth to T on the basis of interpretation of data in which androgen deprivation results in regression of metastatic prostate disease. In this review, we examined data from various studies that investigated the relationship between androgens and EGFR in modulating the growth of normal prostate tissue in animal models and in PCa to develop a better understanding of the potential role of androgens in regulating the growth of normal prostate and loss of this molecular switch in PCa.

A number of studies have demonstrated that androgens promote prostate growth in immature male animals until it reaches its mature size, where it plateaus in the adult animal. Once the mature size of the prostate is reached, it remains quiescent even in the presence of stimulating physiological levels of androgens (Simanainen *et al*, 2007; Wu *et al*, 2007). In PCa, however, epithelial cells develop alternate mechanisms of escaping growth inhibition in the presence of androgens and proliferate, resulting in excessive growth. The mechanisms by which androgens promote prostate cellular growth in the immature animal and at the same time prevent excessive proliferation in the adult animal are thought to involve regulating growth factors and their receptors in the normal prostate (Traish and Wotiz, 1987; St-Arnaud *et al*, 1988; Simanainen *et al*, 2007; Wu *et al*, 2007; Niu *et al*, 2008).

Recently, several studies have demonstrated that ARs in mature prostatic epithelium are critical for maintaining the differentiated phenotype and overall homeostasis of the gland (Simanainen *et al*, 2007; Wu *et al*, 2007; Niu *et al*, 2008). The data suggested that epithelial AR maintains homeostasis through reduced synthesis of proliferation-stimulatory factors or increased synthesis of suppressors of epithelial proliferation. The mechanisms by which AR mediates these processes are complex and may involve specific signalling by paracrine factors (Figure 1). These mechanisms include cross-talk between AR signalling and growth factor receptor pathways (Bonaccorsi *et al*, 2004, 2007). Thus, Léotoing *et al* (2007) proposed that the interaction between these two signalling pathways may be crucial for the acquisition and maintenance of androgen sensitivity in the prostate.

The EGFR signalling pathway is critical in many mammalian cellular systems. Cell survival, growth, proliferation, and differentiation are among the many biochemical and physiological responses to EGFR signalling. EGFR deletion in transgenic animals results in death in the neonatal period because of deficiency in the development of several epithelial organs. Increased EGFR signalling has been associated with progression to invasion and metastasis in tumours. Several studies had related EGFR expression and signalling in the growth of normal prostate and PCa involving a complex signalling pathway that uses multiple downstream modulators (Pfeil *et al*, 2004; Engelman and Cantley, 2008).

Structurally, the EGFR possesses an extra-cellular domain that is involved in specific ligand binding and receptor dimerisation, a single transmembrane domain, and a cytoplasmic domain that hosts intrinsic tyrosine kinase activity. On ligand binding, EGFR forms homo- or heterodimers with other members of the same receptor family, and thereby stimulates cytoplasmic kinase activity, leading to auto- and trans-phosphorylation of tyrosine residues. This phosphorylation results in conformational changes, allowing phospho-tyrosine binding Src homology 2 (SH2)-containing adaptor proteins to bind and initiate a myriad of signalling pathways, which includes the phospholipase C (PLC)-*γ* activated calcium- and PKC-mediated cascades, sarcoma viral oncogene homologue (Ras) and its downstream ERK and JNK pathways, STAT, as well as phosphatidylinositol-3-kinase (PI3K)/Akt. The purpose of this review is to provide a new hypothesis to explain the growth of Pca, irrespective of the androgen environment, because of a molecular switch in regulating EGFR expression by androgens.

## Androgen regulation of expression of EGFR in normal prostate

**Figure 2:**
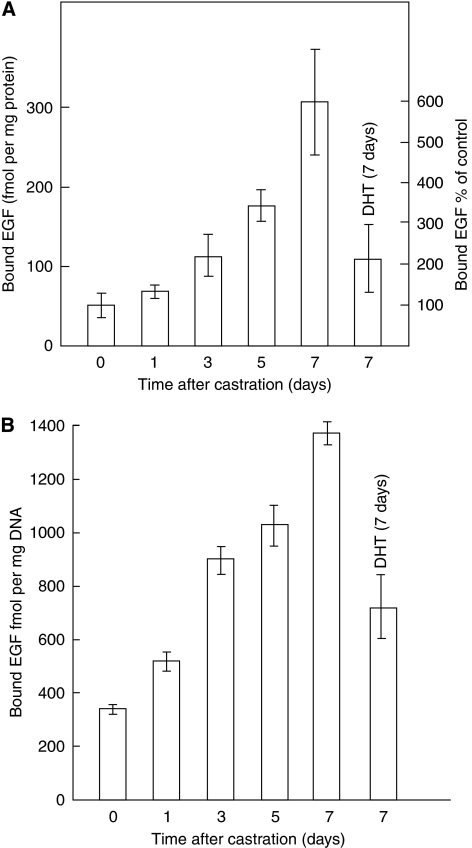
Effects of castration and androgen treatment on EGF binding to prostatic membranes. Castrated animals (five to seven per group) were killed at the times indicated and their prostates were removed; others were treated with 5*α*-DHT (200 *μ*g daily) for 7 days and killed on day 8. Intact animals were killed at the times indicated and their prostates were removed. Prostates from each group were pooled and homogenised, and membranes were prepared and assayed for EGF binding as described. The values shown are the mean of four separate experiments. Upper panel (**A**): data were expressed as femtomoles of EGF binding per mg membrane protein. Lower panel (**B**): data were expressed as femtomoles per mg DNA (adapted from Traish and Wotiz, 1987).

Castration of mature animals produced a time-dependent increase in EGFR protein expression in the prostate (Figure 2) (Traish and Wotiz, 1987; St-Arnaud *et al*, 1988). Treatment of castrated animals with T or 5*α*-DHT reduced EGFR protein expression (Traish and Wotiz, 1987; St-Arnaud *et al*, 1988). The downregulation of EGFR expression is specific to androgens, as oestrogens and progestins did not downregulate EGFR expression. These observations suggested that in the presence of a stimulating environment of androgens, EGFR expression is attenuated to reduce unnecessary excessive growth. In the absence of or because of reduced androgen stimulation, however, EGFR protein expression is increased, probably to prevent complete prostate tissue involution in the androgen-deprived state (Traish and Wotiz, 1987; St-Arnaud *et al*, 1988). Furthermore, medical castration by administration of luteinising hormone-releasing hormone agonist or anti-androgen treatment also increased EGFR protein expression in the prostate (St-Arnaud *et al*, 1988). Nishi *et al* (1996) investigated the changes in the steady-state levels of mRNAs coding for EGFR by northern blot analysis during castration-induced involution, and subsequent re-growth induced by androgen in the prostate. EGFR increased after castration, suggesting that EGFR expression is downregulated by androgens (Nishi *et al*, 1996). Other studies have also shown that EGFR mRNA expression was markedly elevated after castration of 60-day-old adult rats and T treatment reduced EGFR mRNA levels (Itoh *et al*, 1998).

Recent studies also reported that androgen treatment of castrated animals reduced EGFR protein density, diminished epithelial and endothelial cell proliferation, and increased epithelial cell apoptosis (Hammarsten *et al*, 2007). These results suggested that castration-induced increase in EGFR expression may be needed to counterbalance the loss of androgen-stimulated growth and to prevent complete tissue involution (Hammarsten *et al*, 2007). Interestingly, EGFR signalling is inhibited during testosterone-induced growth, which led to growth reduction and prevented excessive proliferation. Treatment of castrated animals with specific monoclonal antibodies against EGFR, such as gefitinib, which inhibit EGFR signalling, led to prostate growth inhibition, suggesting a role for the EGFR signalling pathway in normal prostate growth and in preventing total prostate regression. Inhibition of EGFR signalling with antibody blockade after castration led to a further reduction in epithelial and blood vessel weight. The authors suggested that inhibition of EGFR signalling in castrated animals resulted in rapid prostate involution (Hammarsten *et al*, 2007). These data support the hypothesis that androgens downregulate EGFR expression in normal prostate tissue (Traish and Wotiz, 1987; St-Arnaud *et al*, 1988; Nishi *et al*, 1996).

## Loss of regulation of EGFR expression in PCa by androgens

### Studies in cultured cell lines

Several studies have investigated EGFR expression in PCa cell lines, including LNCaP, PC3, and ALVA101, with mixed results. A number of investigators reported that androgens upregulated EGFR in PCa cell lines (Schuurmans *et al*, 1989; Liu *et al*, 1993; Brass *et al*, 1995; Ravenna *et al*, 1995; Hobisch *et al*, 2004).

Liu *et al* (1993) investigated the proliferation of the androgen-dependent prostate carcinoma cell line ALVA101 in the presence of 5*α*-DHT and EGF, and showed increased cell proliferation and DNA synthesis. It was shown that EGFR ligands increased androgen-induced mitogenesis in the androgen-dependent PCa cell line. EGFR mRNA levels were increased in ALVA101 cells with EGF stimulation. 5*α*-DHT further enhanced EGFR mRNA levels and EGFR protein expression (Liu *et al*, 1993). To support the contention that increased EGFR expression mediates androgen-induced cell proliferation, an EGFR-directed antibody was shown to be effective in inhibiting 5*α*-DHT-induced mitogenesis. These observations suggest that androgen stimulation enhanced EGFR receptor activation with EGF in PCa cells. In contrast to that of the normal prostate, in which androgens decrease EGFR expression, in ALVA101 cells androgen-induced EGFR expression resulted in increased cell division and upregulation of EGFR, thus enhancing proliferation of PCa cells (Liu *et al*, 1993).

**Figure 3:**
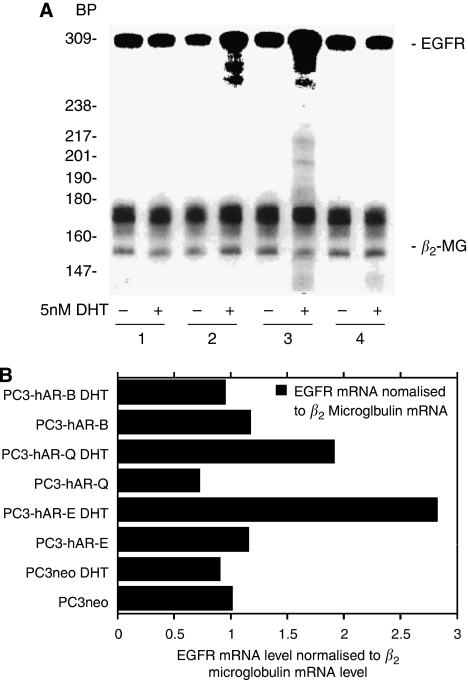
(**A**) Ribonuclease protection assay using ^32^P-labelled antisense EGFR and b2-microglobulin RNA riboprobes to assess the effect of 5 nM DHT on EGFR expression in stable PC3 transfectant cells. Total RNA was isolated from PC3neo (1), PC3- hAR-Q (2), PC3-HAR-E (3), and PC3-HAR-B (4) cells grown in the presence (+) or absence (−) of 5 nM DHT for 48 h. BP, nucleotide bp markers are ^32^P-end-labelled DNA fragments of the PBR322 vector restricted with Mspl. (**B**) PhosphorImager analysis of EGFR expression normalised to b2 microglobulin expression (adapted from Brass *et al*, 1995).

Brass *et al* (1995) further investigated the role of androgens in PCa by re-introducing AR into PC3 cell lines. EGF and 5*α*-DHT treatment of PC3-AR cells stimulated growth independently of each other. When added together, they acted synergistically to induce a greater than four-fold increase in proliferation. 5*α*-DHT treatment induced a two-fold increase in EGFR transcription and about a 50% increase in EGFR protein levels. EGF binding to EGFR in PC3-AR cells exposed to 5*α*-DHT was increased, suggesting an upregulation of EGFR expression by androgens (Figure 3).

Contrary to the above findings, Sherwood *et al* (1998) showed that high levels of EGFR expression and phosphorylation were observed only in androgen-independent human PCa cell lines, PC3 and DU 145, but not in LNCaP cell lines, which are androgen sensitive. Cinar *et al* (2001) showed that human PCa cells transfected with hAR exhibited reduced growth, invasion, and migratory behaviour *in vitro* and tumour growth *in vivo*. Bonaccorsi *et al* (2004, 2007) investigated EGF-activated signalling in PC3 before and after transfection with AR. The authors showed that PC3 transfected with AR had significant reduction in EGFR autophosphorylation and PI3 kinase activation, suggesting that androgens modulate EGFR expression and function. It was proposed that re-constitution of the AR in androgen-independent PCa cell lines leads to a less-malignant phenotype through an AR-EGFR cross-talk.

Although studies with PCa cells offer an experimental model to study the relationship between androgens and EGFR expression and function *in vitro*, we must be aware that cell lines undergo changes because of culture, storage, and experimental manipulation conditions. Thus, the *in vitro* cell culture data must be viewed with caution.

### Studies in prostate tumour tissue specimen

Di Lorenzo *et al* (2002) investigated EGFR expression in PCa tissue from 58 patients. Twenty-nine of these patients had primary tumours and were treated with radical prostatectomy as first-line therapy. EGFR expression was positive in 12 (41.4%) of the 29 patients. In a second group of patients (29) with primary tumours, the investigators first treated patients with ADT and with anti-androgens before radical prostatectomy. EGFR expression was found in 22 (75.9%) of 29 patients. A third group of 16 patients with metastatic disease and hormone-refractory PCa were found to be all positive for EGFR expression (100%). These data suggest that ADT increased the expression of EGFR in human PCa. The authors also demonstrated a significant association between EGFR expression and higher Gleason scores and PSA levels. EGFR expression significantly correlated with disease relapse in patients. The authors further showed that 23 of 34 patients with EGFR-positive tumours had a disease recurrence (67.0%), compared with 2 of 24 patients (8.3%) who had EGFR-negative PCa. EGFR expression represented the only independent parameter that was significantly associated with disease relapse when compared with the Gleason score to T and N status and to c-erbB-2 expression.

It should be noted that, in this study (Di Lorenzo *et al*, 2002), the AR content or distribution within these tissue specimens was not assessed in any of the tumour specimens investigated. Thus, no direct correlation between EGFR expression and AR could be determined from these observations. This represents a limitation to the conclusions of this study.

The studies described above support a role for androgens in regulating EGFR expression in the development of PCa and, more specifically, in the progression to an androgen-independent, hormone-refractory clinical behaviour. Hernes *et al* (2004) also investigated prostatic tissue biopsies from patients before ADT and during androgen-independent PCa development. The data revealed that EGFR expression is significantly increased with the development of androgen independence. Shah *et al* (2006) demonstrated that EGFR expression in prostate tumour tissues was strongly associated with the hormone-refractory status. Scher *et al* (1995) showed a homogeneous staining pattern for EGFR in 17 of 19 androgen-independent refractory metastatic PCa specimens. A similar increase in EGFR expression has been demonstrated in a smaller series of PCa patients: untreated, hormone naive (15% positive), hormone responsive (35% positive), and hormone refractory (48% positive) (Gil-Diaz de medina *et al*, 1998). These observations provide bases for cross-talk between the EGFR family and AR-activated pathways in PCa.

The results from studies with normal prostate tissues (Traish and Wotiz, 1987; St-Arnaud *et al*, 1988; Nishi *et al*, 1996; Itoh *et al*, 1998; Hammarsten *et al*, 2007) and those described above in cell culture studies and human prostate tumour tissue biopsies support the concept that EGFR-stimulated growth of PCa escaped androgen control because of a molecular switch in the cross-talk pathway between androgens and EGFR.

## Discussion

In normal human prostate tissues, EGFR expression is under negative androgen regulation. However, in PCa, EGFR expression is upregulated, suggesting an increased expression in advanced cancer (Di Lorenzo *et al*, 2002), and correlated with a high Gleason score and tumour progression from an androgen-dependent to an androgen-independent state (Sherwood and Lee, 1995; Syed and Tolcher, 2003). Miyamoto *et al* (2008) suggested that EGFR and bFGF were downregulated in androgen-sensitive cells expressing AR, in which, as in androgen-independent cells, these receptors were upregulated. Expression of EGFR has been shown to correlate with disease relapse and/or progression to androgen-independent disease in patients with PCa.

Cross-talk between the AR signalling pathway and growth factor signalling may represent a key pathway during prostate PCa progression, which may confer a survival and invasion advantage to PCa (Ravenna *et al*, 1995; Miyamoto *et al*, 2008; Zhu and Kyprianou, 2008). Zhu and Kyprianou (2008) reviewed the cross-talk between growth factor receptors and AR in regulating prostate cell differentiation, proliferation, apoptosis, and survival. These include IL 6 R, EGFR, TGF*β*R1 and TGF*β*R2, IGFR, FGFR, and VEGFR. Cross-talk between tyrosine kinase receptors (TKR) and AR takes place via specific and overlapping pathways (Cai *et al*, 2009). Furthermore, sensitisation of the androgenic response by multi-functional growth factor signalling pathways is thought to be one of the mechanisms through which AR contributes to the emergence of androgen-independent prostate tumours. The significance of this altered cross-talk in PCa progression is of clinical importance in understanding the pathology of the disease.

It has been suggested that regulation of EGFR by androgen is disrupted in PCa (Russell *et al* (1998) and that 5*α*DHT treatment increased EGFR mRNA and protein levels in LNCaP cells (Pignon *et al*, 2009)). EGFR family members have a critical function in the proliferation, migration, survival, and differentiation of target cells. Dysregulation of signalling by ErbBs has been implicated in the pathogenesis and progression of human cancers (El Sheikh *et al*, 2003). The continued expression of AR and AR-regulated genes in androgen-independent PCa (AI PC) suggests that alternative signalling pathways are used to activate AR. Elements of the AR and ErbB pathways interact, cross-over, and converge on targets downstream of each other's signalling cascades to promote AI PC cell survival.

In addition to the EGFR family, an Eph receptor family of tyrosine receptor kinases (RTK) have critical functions in normal growth and development but were shown to be overexpressed in a host of human cancers (Zeng *et al*, 2003; Surawska *et al*, 2004; Fox *et al*, 2006; Taddei *et al*, 2009). Eph receptors are thought to modulate interactions of cells within the tumour and between tumour cells. They also regulate stroma vasculature interaction and are implicated in PCa progression (Zeng *et al*, 2003; Fox *et al*, 2006; Taddei *et al*, 2009). As limited data are available on the interaction between AR and Eph receptors in androgen-sensitive and independent tumours, we will not discuss this subject further in this review.

Recently, Morgentaler and Traish (2009) challenged the belief that PCa growth is dependent on T levels and a saturation model is proposed that accounts for the seemingly contradictory results in human PCa studies (Morgentaler and Traish, 2009). It was suggested that the ability of androgens to stimulate PCa growth is limited and may involve other biochemical factors. A conceptual framework to account for the dramatic effects of castration, as well as the minor impact of T administration, was presented. Here, we provide additional evidence of the potential possibility that androgens regulate the receptor of one of the growth factors that stimulate prostate growth, and loss of androgen regulation in PCa may be one of the factors that contribute to the androgen independence of PCa.

Normal prostate cells express AR, and androgens are required for the growth and function of normal prostate. During neoplastic transformation of prostatic epithelial cells into tumour cells, expression of AR is retained by tumour cells, which is responsible for androgen-dependent tumour growth. The fact that tumour cells retained the ability to express AR implicated androgens in PCa growth. However, to date, no mechanism has been delineated to suggest that androgen signalling *per se* is responsible for the development of prostate tumours. Indeed, in the prostate, tumour growth continues to depend on androgens similar to that of normal epithelial cells. However, at some point in time, tumour cells become androgen independent and grow in the absence of androgens (androgen refractory). The transition from androgen dependence to androgen independence is most often accelerated by ADT.

Similar to the role of androgens in PCa, oestrogens regulate the expression and activity of EGFR in breast cancer. Arpino *et al* (2008) reviewed the cross-talk between oestrogen receptor (ER) and the HER tyrosine kinase receptor family, and proposed a mechanism of resistance to endocrine therapy in breast cancer. Inhibition of ER expression or oestradiol deprivation or treatment with anti-oestrogen increased EGFR expression in breast cancer cell lines, suggesting a complex control mechanism (Lichtner, 2003; Osborne *et al*, 2005) An oestrogen-independent growth ensues through alternative mechanisms during oestrogen deprivation or anti-oestrogen treatment. Upregulation of EGFR in response to oestrogen depletion was considered to be one of the survival mechanisms of tumour cells (Yarden *et al*, 2001). Thus, in ER-positive breast cancer cells, oestrogen is actively involved in the suppression of EGFR expression, whereas in ER-negative tumours, EGFR expression is increased. These findings suggest that progression of breast cancer from hormone dependence to hormone independence may involve upregulation of EGFR.

In normal prostate tissue, androgens downregulate EFGR expression (Figure 1, reaction 7). The downregulation of EGFR by androgens in normal cells may be responsible for the inhibition of excessive growth in normal prostate. In PCa, however, androgens seem to upregulate EGFR expression (Figure 1, reaction 7) by a molecular switch, which remains to be defined. Loss of androgen downregulation of EGFR expression in PCa increases EGFR receptor expression and signalling and may produce excessive cellular proliferation.

Transition of the prostatic epithelium from androgen dependence to androgen independence may occur by clonal selection from a heterogeneous population of androgen-dependent cells during androgen deprivation. Alternatively, precursor stem cells may adapt to an androgen-deprived environment and differentiate in the absence of androgens. Another possibility is that, in the castrated patient, adrenal androgens may represent sufficient androgens to stimulate androgen-responsive cells (Russell *et al*, 1998).

One of the potential mechanisms is the activation of AR by phosphorylation in the absence of the hormone, through the EGFR downstream signalling pathway. Although ADT initially reduces tumour growth, it often results in an increased EGFR expression and subsequently increased androgen-induced growth and development of androgen-refractory tumours and metastasis. Thus, increased EGFR expression and signalling with androgen deprivation may be responsible for the transition of tumours from androgen dependence to androgen independence. Another mechanism that may influence the expression and activity of EGFR is homodimerisation and heterodimerisation of EGFR with receptor subunits of members of the EGFR family (Hughes *et al*, 2009; Patel *et al*, 2009). This reaction may be significantly altered in PCa cells.

Several limitations of this hypothesis should be acknowledged. First, PCa aggressiveness, androgen sensitivity, and histological appearance exhibit marked variations and no single specific experimental model mimicked such various facets of the clinical disease. Second, because most of the studies were carried out with cell lines including LNCaP, which demonstrate androgen sensitivity but not androgen dependence, the concern remains that limitations exist on interpretation of data from studies using cell lines for understanding the molecular basis of PCa (Russell *et al*, 1998). Furthermore, EGF has been shown to activate the transcriptional activity of AR by increasing the expression or activity of AR co-activators in PCa cells and, in this way, is thought to promote malignant progression and metastasis of advanced PCa. Third, the cross-talk between AR and the different signalling pathways is complex, and at present poorly understood. Given the complexities of such interactions and the heterogeneity of PCa, a detailed understanding of these processes is necessary to develop appropriate therapeutic strategies for patients with PCa. Therefore, we acknowledge that this review is focused on single gene hypothesis, which, in view of the complexities of the molecular interactions between AR pathways and other signalling molecules, may limit broader interpretations.

It has been shown that overexpression of EGFR in 3T3-L1 adipocytes (200 000–250 000 receptors per cell) confers EGF-inducible GLUT4-mediated glucose uptake (Van Epps-Fung, 1996). Glucose transport and phosphorylation were increased significantly in MDA–MB-468 breast cancer cells expressing a high number of EGFR (Kaplan *et al*, 1990). In gefitinib-sensitive cell lines, the EGFR kinase inhibitor produced a dramatic decrease in fluoro-deoxyglucose (FDG) uptake, and glucose transport rates were reduced by 2.6-fold. This was associated with a translocation of glucose transporters (GLUT3) from the plasma membrane to the cytosol. In contrast, gefitinib-resistant cells exhibited no measurable changes in FDG uptake, either in cell culture or *in vivo* (Su *et al*, 2006). Recent findings (Weihua *et al*, 2008) have suggested that overexpression of EGFR is associated with tumour cell proliferation and survival, and this was attributed to maintaining the intracellular glucose level through interaction and stabilisation of the sodium/glucose cotransporter 1 (SGLT1). This suggests that inhibition of the EGFR kinase activity for cancer therapy may not provide the expected efficacy (Weihua *et al*, 2008). Thus, it is possible that in PCa, and in the absence of androgens, EGFR is overexpressed in prostate tumours facilitating glucose transport into cells by associating with and stabilising an SGLT1 without requiring EGFR kinase activity as proposed by Weihua *et al* (2008). The interaction between EGFR and SGLT1 may represent a novel mechanism of adaptation by cancer cells to meet energy demand for cellular growth and suggests that EGFR contributes to increased cell survival and metastasis independent of the EGFR signalling pathway. This novel mechanism may be exploited in targeting EGFR expression without targeting the kinase *per se* to develop new treatment strategies for the management of androgen-independent PCa.

In view of this novel concept, an increased EGFR expression in PCa because of loss of androgen-dependent regulation would enhance tumour growth, invasion, and metastasis due to increased EGFR in tumour cells. Thus, the interplay between AR and EGFR in regulating cellular growth of the prostate is of paramount importance. Any molecular switch that disrupts the regulation of EGFR expression by androgens and the mechanisms involved in mediating the cross-talk between AR and EGFR would result in loss of cellular regulation and promote uncontrolled growth. The loss of regulation of EGFR expression by androgens in PCa represents an important pathway in cellular growth, invasion, and metastasis. A better understanding of this regulation may represent a target for therapeutic management of PCa.
